# Multifocal Brucellosis in a Young Patient: When a Non-inflammatory Knee Effusion Hides Systematic Complications

**DOI:** 10.7759/cureus.81088

**Published:** 2025-03-24

**Authors:** Assadullah Dahani, Maryam AlNuaimi, Ajith C Thomas, Hazem E Hussein, Jithin Jameel

**Affiliations:** 1 Internal Medicine and Rheumatology, Ibrahim Bin Hamad Obaidullah Hospital, Emirates Health Services, Ras Al Khaimah, ARE; 2 Internal Medicine, Ibrahim Bin Hamad Obaidullah Hospital, Emirates Health Services, Ras Al Khaimah, ARE

**Keywords:** brucellosis, hepatic microabscess, knee pain, non-inflammatory effusion, osteomyelitis

## Abstract

Brucellosis, a prevalent zoonosis transmitted through unpasteurized dairy products or contact with infected animals, typically presents with fever, malaise, fatigue, hepatic involvement, and various osteoarticular manifestations. However, it rarely presents with hepatic microabscesses and osteomyelitis. We report a case of a 26-year-old female with no known comorbidities who presented with complaints of fever and right knee swelling for three days. On clinical assessment, her pulse was 120 beats per minute, respiratory rate was 18 breaths per minute, blood pressure was 80/54 mmHg, and temperature was 38°C. Musculoskeletal examination revealed knee effusion only. The systemic examination was unremarkable except for an innocent murmur in the heart. Laboratory results showed hemoglobin 6.7 g/dL, mean corpuscular volume 60, white blood cell count 3.29, platelets 232, erythrocyte sedimentation rate 40, C-reactive protein 53 mg/dL, and mild derangement in liver function tests. Autoimmune workup was negative. Synovial fluid analysis showed a non-inflammatory effusion. A computed tomography (CT) scan of the abdomen revealed multiple small microabscesses. The patient was started on empirical antibiotics based on the suspicion of a liver abscess and symptomatic treatment for joint pain, but her symptoms did not improve. Magnetic resonance imaging (MRI) of the knee suggested joint effusion and osteomyelitis. Brucellosis was diagnosed based on antibodies and blood culture. She was started on standard treatment, her symptoms improved, and she was discharged with a six-week course of treatment.

Our case highlights the diagnostic challenges of brucellosis, a zoonotic infection with diverse manifestations, in a young female presenting with fever, non-inflammatory knee effusion, osteomyelitis, and hepatic microabscesses. It underscores the importance of a thorough workup and cross-specialty collaboration, including rheumatology, infectious diseases, and radiology, to avoid misdiagnosis and ensure timely intervention.

## Introduction

Brucella is a gram-negative coccobacillus, non-spore-forming, and non-motile bacterium. There are five species of Brucella, three of which are of primary importance in humans: Brucella melitensis, Brucella abortus, and Brucella suis. Brucellosis is a zoonotic disease transmitted through the handling of infected animals such as goats, sheep, swine, and cows, or through the consumption of contaminated dairy products. Clinical manifestations include malaise, fatigue, fever, joint pain, arthralgia/arthritis, backache, and hepatosplenomegaly. Diagnosis is primarily based on blood culture and serology. Osteoarticular manifestations may include septic arthritis, reactive arthritis, osteomyelitis, spondylitis, and sacroiliitis. Liver involvement is common and may lead to elevated transaminases, mild hepatosplenomegaly, and, in rare cases, acute hepatitis or liver failure. Rovary et al. also reported the formation of liver abscesses (brucelloma), a very rare complication usually seen in chronic brucellosis, but it can also occur in acute infection [1].

## Case presentation

**Figure 1:**
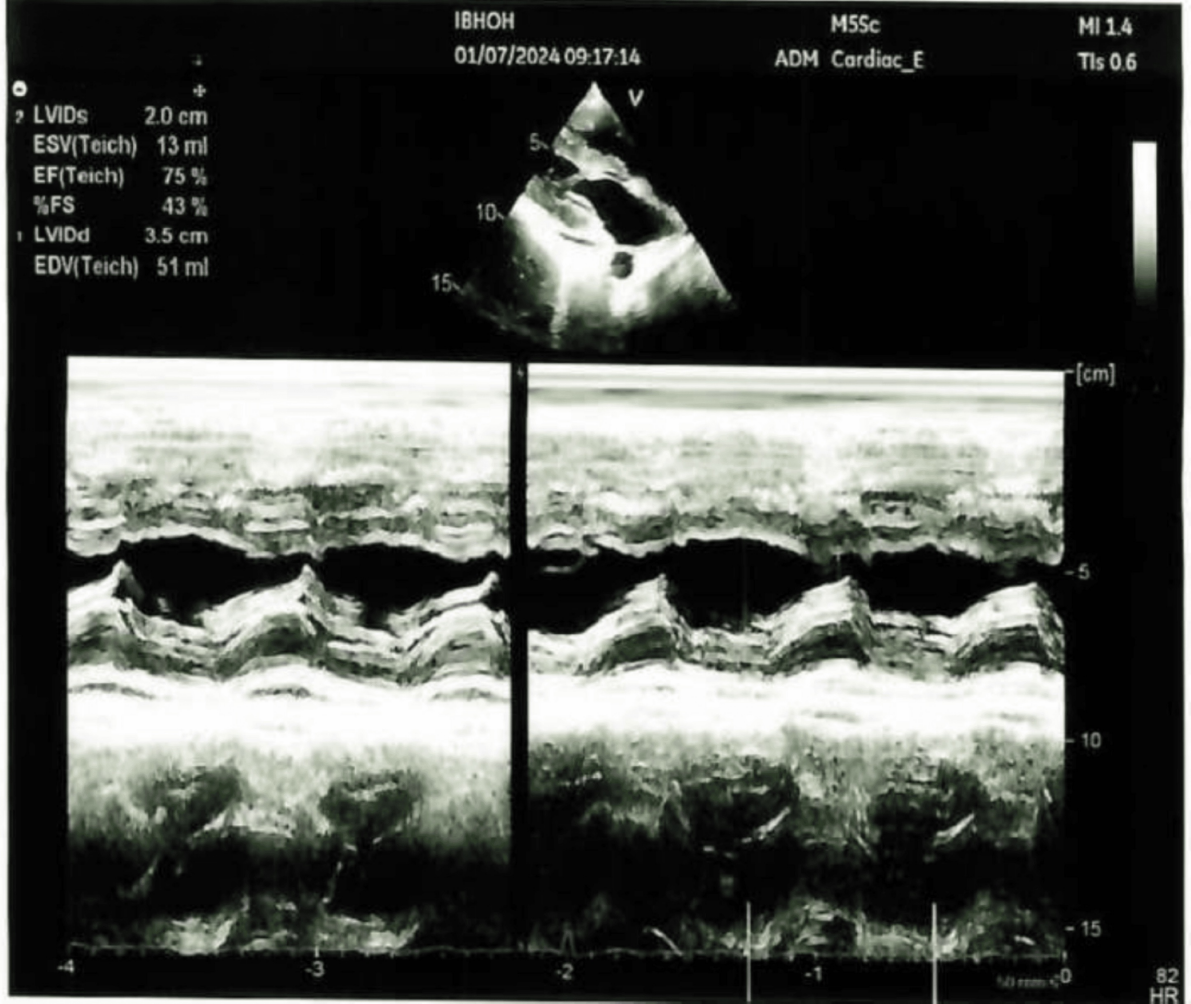
Echocardiography No abnormality detected.

**Figure 2:**
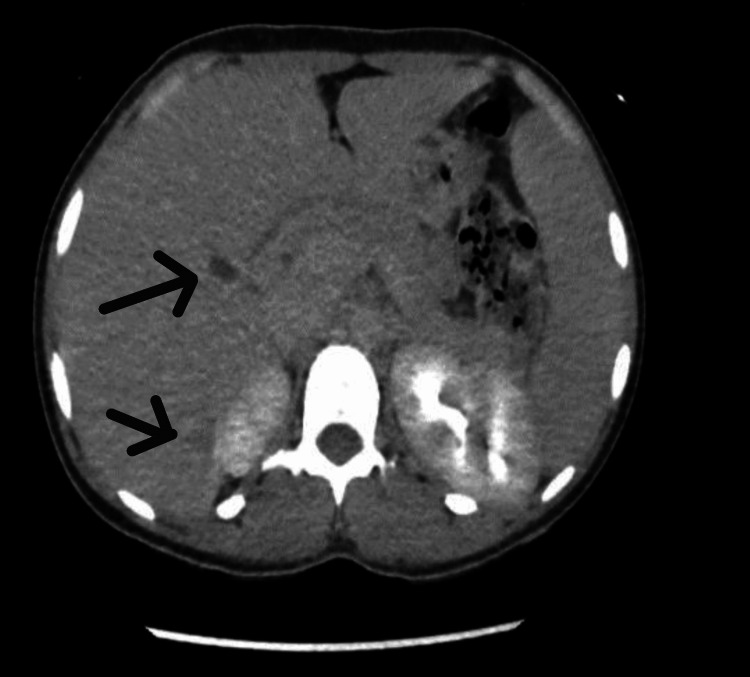
CT abdomen: hepatic microabscesses CT, computed tomography

**Figure 3:**
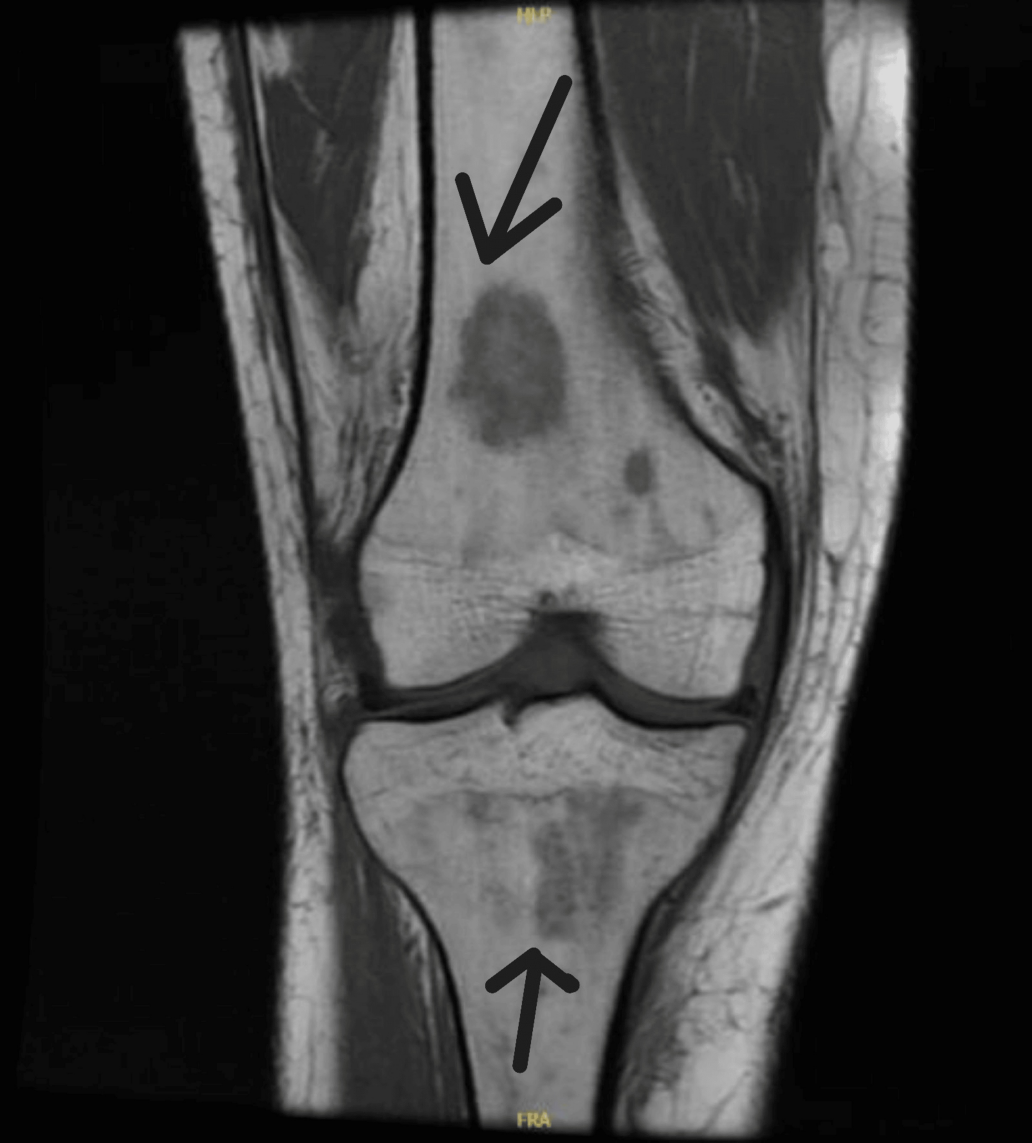
MRI knee: marrow signal abnormalities involving the distal femur and proximal tibia MRI, magnetic resonance imaging

**Figure 4:**
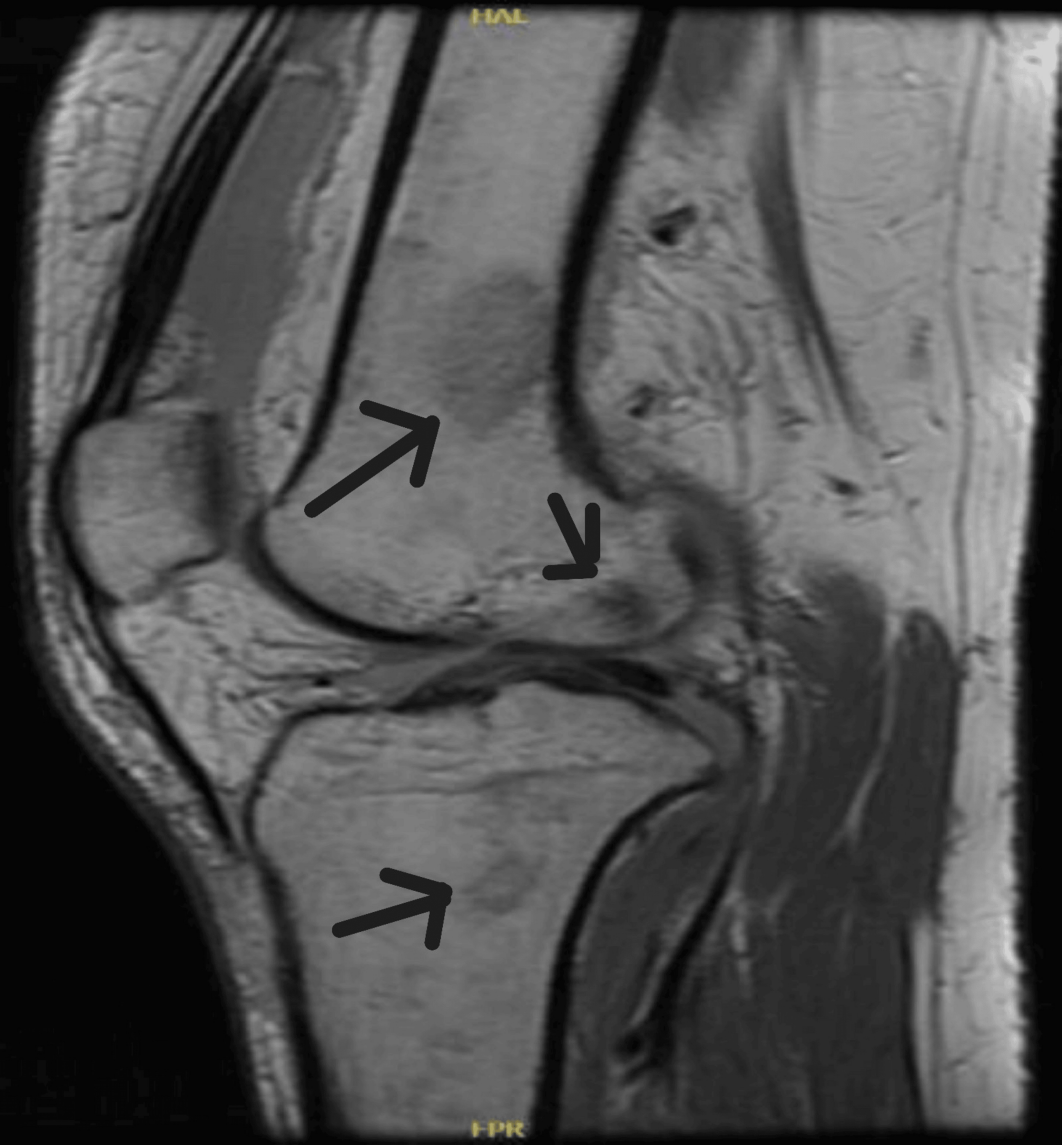
MRI knee: marrow signal abnormalities involving the distal femur and proximal tibia MRI, magnetic resonance imaging

**Figure 5:**
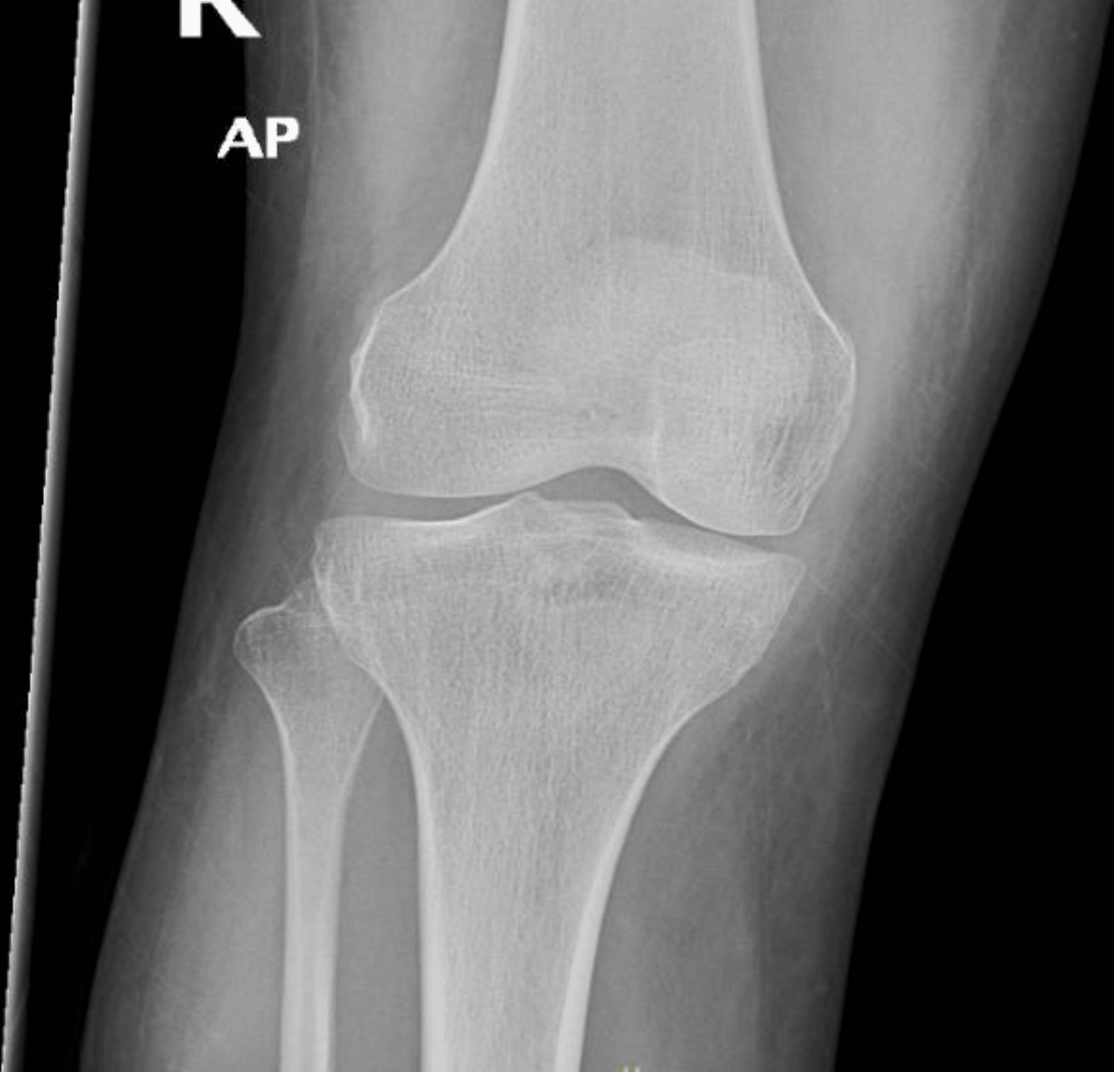
X-ray knee: normal

We present a case of a 26-year-old female who presented with a high-grade fever for three days, accompanied by right knee swelling. There was no history of weight loss, pain in other joints, trauma, rash, backache, shortness of breath, cough, or genitourinary or gastrointestinal symptoms. She denied any history of direct contact with animals but consumed milk regularly. The knee pain was severe enough to impair her ability to walk. Examination of the other joints and back was unremarkable. No rash, oral ulcers, alopecia, or enthesitis were observed. Cardiovascular examination revealed an innocent systolic murmur (2/6). Abdominal and respiratory examinations were normal. Laboratory results included hemoglobin 6.7 g/dL, mean corpuscular volume 60 fL, white blood cell count 3.29×10⁹/L, platelet count 232×10⁹/L, erythrocyte sedimentation rate 40 mm/h, and C-reactive protein 53 mg/L (Table 1). Further workup of low hemoglobin revealed iron deficiency anemia. Autoimmune workup was negative. Liver function tests revealed elevated alkaline phosphatase and mildly raised aspartate aminotransferase levels. Transthoracic and transesophageal echocardiography were normal (Figure 1). Synovial fluid analysis was non-inflammatory with 232 WBC/mm³, and both Gram stain and culture were negative. Preliminary blood cultures showed no growth. A computed tomography (CT) scan of the abdomen revealed multiple small hepatic microabscesses (Figure 2). Magnetic resonance imaging (MRI) of the knee revealed marrow signal abnormalities in the distal femur and proximal tibia, along with moderate knee effusion (Figure 3 and Figure 4). X-ray of the knee (Figure 5) was normal, but CT scan imaging revealed moderate right knee effusion. The patient was started on antibiotics for a presumed liver abscess and symptomatic treatment for joint pain, but her symptoms did not improve. Brucella IgM antibodies were 1:640, and blood culture revealed slow growth of gram-negative coccobacilli on the fifth day, later identified as Brucella melitensis on the seventh day. The organism was found to be sensitive to gentamicin, doxycycline, and rifampin, according to the regional brucellosis treatment standard. The patient was treated with gentamicin and doxycycline for two weeks, followed by doxycycline and rifampin for six weeks. She was given follow-up care in the outpatient department for further assessment and extension of antibiotic therapy. After five days of antibiotic therapy, she became afebrile, and her knee pain improved.

**Table 1:** Laboratory results

Laboratory	Result	Reference range
Hemoglobin	6.5 g/dL	11.6-16.6g/dL
Mean corpuscular volume	60 fL	80-100 fL
White blood cell	3.29x10^9^/L	4.0-11x10^9^/L
Platelets	232x10^9^/L	150-400x10^9^/L
Erythrocyte sedimentation rate	40 mm/hr	<15 mm/hr
C-reactive protein	53 mg/dL	<5 mg/dL
Bilirubin	1.0 mg/dL	0.3-1.3 mg/dL
ALT	30 U/L	<55 U/L
AST	70 U/L	<48 U/L
Alkaline phosphate	250 U/L	40-129 U/L
Urea	10 mg/dL	6-24 mg/dL
Creatinine	0.8 mg/dL	0.7-1.1 mg/dL
ANA	1:40	>1:80
DsDNA	Negative	Positive
Rheumatoid factor	Negative	Positive
Anti-CCP	Negative	Positive
Synovial fluid white blood cell count	232 WBC/mm³	<200 WBC/mm³
Synovial fluid gram staining	Organism	No organism
Synovial fluid culture	No organism	No growth
Blood culture	Brucella	No growth
Brucella antibodies	1:640	<1:80

## Discussion

Brucellosis is a zoonotic disease prevalent in West Asia, the Middle East, India, Southern Europe, and Latin America. Laine CG et al. reported an increasing global prevalence, with an estimated 1.2 to 1.6 million new cases annually [2,3]. According to available data, the incidence of human brucellosis in the United Arab Emirates (UAE) is estimated to be 41 cases per million people per year. Brucellosis affects the immune system, leading to chronic infection and a wide range of clinical symptoms, from fever and joint pain to more severe manifestations such as endocarditis and neurological involvement. In our case, the patient presented with fever and joint pain but notably did not report right hypochondrial or abdominal pain. However, imaging revealed hepatic abscesses, which were further supported by blood cultures and Brucella antibodies. Hepatic abscesses are a rare complication of brucellosis, though they have been documented in the literature [1] and are referred to as brucelloma. Williams RK et al. and Pérez SD et al. also found that it can occur in both acute and chronic forms [4] and can be either single or multiple [5]. In our case, the patient had multiple abscesses.

Hasanjani Roushan et al. also found that osteoarticular involvement occurs in 10% to 85% of brucellosis cases [6]. Common manifestations include peripheral arthritis, sacroiliitis, osteomyelitis, tenosynovitis, and bursitis. Axial involvement is more common than peripheral arthritis and can affect the knee, ankle, wrist, shoulder, and interphalangeal joints. The knee, hip, and ankle joints are among the most frequently involved peripheral regions. Monoarthritis is more commonly seen in children, typically affecting the hip or knee, with concurrent osteomyelitis of the adjacent bones. In our patient, knee pain and swelling were present, and synovial fluid analysis was non-inflammatory. MRI suggested osteomyelitis involving the distal femur and proximal tibia. Although osteomyelitis is more common in the vertebral region, it can also occur in the femur, tibia, and other bones. Nicola Kavanagh et al. reported that acute osteomyelitis is chiefly caused by hematogenous spread in adults [7].

Blood culture positivity for Brucella can vary depending on the method used and the duration of the disease. In our patient, blood cultures were positive, and Brucella melitensis was identified as sensitive to gentamicin, doxycycline, and rifampin. Brucella IgM also showed positive results with high titers. The drugs commonly used in treatment include doxycycline, rifampin, streptomycin, gentamicin, trimethoprim/sulfamethoxazole, and ciprofloxacin. Combination therapy is associated with better treatment outcomes and fewer relapses. Bayindir Y et al. found that a triple therapy regimen is more effective in treating complicated infections [8]. The patient was treated with gentamicin and doxycycline for two weeks, followed by doxycycline and rifampin for six weeks. Our patient also received gentamicin and doxycycline initially, and later continued with doxycycline and rifampin, as the patient responded well. Continuation of gentamicin would have been more effective, but there was a risk of side effects from the drug with longer use. Therefore, gentamicin was discontinued since the patient had already responded well. After 5 days of treatment, she became afebrile, and her knee pain improved. The patient was consulted via electronic consultation after the third week of discharge and reported no recurrence of symptoms.

## Conclusions

This case highlights the diagnostic challenges of brucellosis, a zoonotic infection with diverse and protean manifestations, in a young female presenting with fever, non-inflammatory knee effusion, osteomyelitis, and hepatic microabscesses. It emphasizes the importance of a thorough workup and cross-specialty collaboration between rheumatology, infectious disease, and radiology to avoid misdiagnosis and ensure timely intervention to prevent morbidity and mortality.
